# A Novel Type of Colony Formation in Marine Planktonic Diatoms Revealed by Atomic Force Microscopy

**DOI:** 10.1371/journal.pone.0044851

**Published:** 2012-09-20

**Authors:** Sunčica Bosak, Galja Pletikapić, Amela Hozić, Vesna Svetličić, Diana Sarno, Damir Viličić

**Affiliations:** 1 Divison of Biology, Faculty of Science, University of Zagreb, Zagreb, Croatia; 2 Division for Marine and Environmental Research, Ruđer Bošković Institute, Zagreb, Croatia; 3 Stazione Zoologica Anton Dohrn, Naples, Italy; Dalhousie University, Canada

## Abstract

Diatoms have evolved a variety of colonial life forms in which cells are connected by organic threads, mucilage pads or silicate structures. In this study, we provide the first description of a novel strategy of colony formation among marine planktonic diatoms. *Bacteriastrum jadranum* forms loose but regular chains with distinct heterovalvate terminal cells. The colonial cells and their siliceous projections, the setae, are not in direct contact; instead, they are enclosed within the optically transparent organic matrix. This cell jacket structure was detected by staining procedure with Alcian Blue, which showed that the polysaccharides are predominant matrix constituents and revealed that the jacket reaches the span of the setae. The scanning electron microscopy (SEM) observations showed distinguishable fibrillar network firmly associated with cells. Using atomic force microscopy (AFM), we were able to visualise and characterise the cell jacket structure at molecular resolution. At nanoscale resolution, the cell jacket appears as a cross-linked fibrillar network organised into a recognisable structure. The circular patches of self-repeating pattern (hexagonal pores with openings of 8–100 nm) are connected through thicker surrounding fibrils and reinforced by branching fibrils. The pore-forming fibrils within the patches are only 0.6–1.6 nm high, the surrounding fibrils connecting patches are 2.0–2.8 nm high, and the branching fibrils are considerably wider but not higher than 4.0 nm. The discovered polysaccharide fibrillar network is highly organised and delicately structured with a monomolecular fibril height of 0.6 nm. We conclude that the *Bacteriastrum* polysaccharide jacket represents an essential part of the cell, as the conjunction of the polymer network with the frustule appears to be extremely tight and such specific and unique patterns have never been found in self-assembled polysaccharide gel networks, which are usually encountered in the marine environment.

## Introduction

Diatoms (Bacillariophyceae) are unicellular microalgae that account for as much as 20% of global photosynthetic carbon fixation estimated by their abundance in marine plankton [1]. Although diatom cells mainly function as autonomous entities, in many species, sibling cells adhere to each other to create various types of colonies. Different forms of colonial lifestyles in diatoms are considered evolutionary adaptations by species to diverse ecological and physiological requirements. Cells connect to form colonies by a variety of means, such as mucilage pads or stalks, chitin threads and/or different types of siliceous structures [2]. In species within the planktonic centric diatom family Chaetocerotaceae [3], cells typically form inseparable chain colonies by interlocking/fusion of the siliceous hollow spine-like projections, called setae, protruding from the valve margin. The family is comprised of two genera, *Chaetoceros* and *Bacteriastrum*; the main character difference is the number of setae linking the sibling cells: two setae per valve in the former genus and numerous setae (6–20) in the latter genus [2].

The marine planktonic diatom *Bacteriastrum jadranum* was firstly observed in planktonic assemblages from the Adriatic Sea [4] and recently described by Godrijan et al. [5]. Unlike all other known colonial *Bacteriastrum* species [6], the chain formation does not involve the fusion of the setae of adjacent cells. Moreover, no thread or any other organic or inorganic substance clearly visible by light or electron microscopy connects the cells. These observations stimulated more detailed morphological analyses aimed at visualising the possible existence of an optically transparent matrix presumably keeping the cells in chain. We used the high resolution imaging technique of atomic force microscopy (AFM), together with more common scanning electron microscopy (SEM) and staining techniques, to verify the presence of an extracellular polymer substance (EPS), which is the most obvious and probable matrix constituent. EPS can be imaged and characterised down to the molecular scale while attached to the diatom cell in a near-natural environment using AFM, as recently shown in a study of the marine pennate diatom *Cylindrotheca closterium* EPS [7], [8]. The AFM imaging introduced in this study provided the first evidence that *Bacteriastrum jadranum* cells are enclosed in a fibrillar polysaccharide network. This finding prompted further experiments using light microscopy after polysaccharide specific staining and SEM visualization on the whole cells. The results of our investigations revealed that this diatom species possesses a unique method of colony formation by enclosing the cell chains in a well-defined polysaccharide network with fibrils organised in self-repeating patterns. This strategy represents the true novelty previously unrecorded, not only for the whole family Chaetocerotaceae but also for planktonic marine diatoms in general.

## Results and Discussion

### Morphology of *Bacteriastrum jadranum* colony

**Figure 1:**
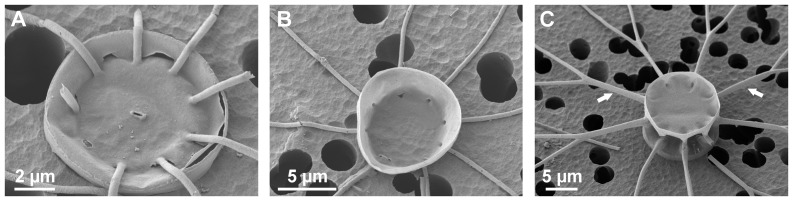
Valve and setae morphology in *Bacteriastrum jadranum* and *B. furcatum.* SEM images of (A) *B. jadranum* terminal valve with a central slit-shaped process (B) single intercalary *B. jadranum* valve lacking central slit-shaped process. Note that setae do not fuse with other setae (C) two sibling valves of the intercalary cells of *B. furcatum* connected by the fused setae (indicated by arrows).

**Figure 2:**
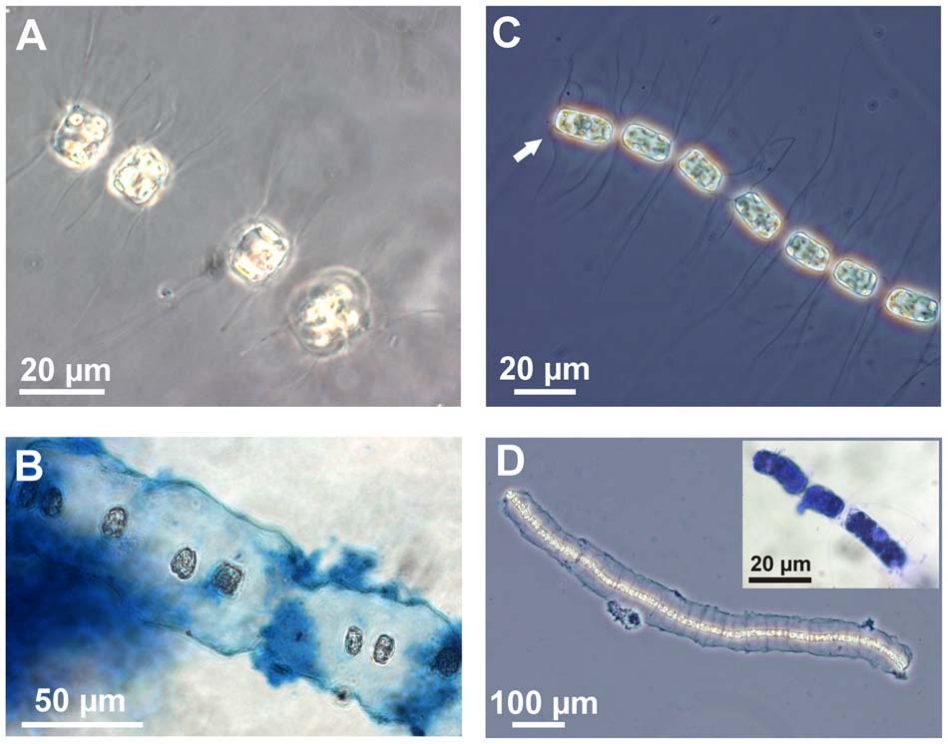
Morphology of *Bacteriastrum jadranum* chain colony. Light micrographs of *B. jadranum*. A–B observations from field sample. (A) Two pairs of recently divided cells in girdle view. The newly formed valves can be recognised by the shorter distance between them and the presence of shorter setae. (B) The middle part of the chain colony enclosed within the Alcian Blue stained structure. The distance between cells extends up to 4 times longer than the cell diameter. C–D observations from culture, phase contrast (C) A Terminal part of the colony in girdle view. Note the different morphology of the terminal setae (indicated by the arrow). (D) A complete colony enclosed within the cell jacket visualised by Alcian Blue staining. The chain colony appears more compact probably due to the peculiar hydrodynamics in culture conditions that result in a lack of cell movement usually present in the natural environment. Coomassie Blue G -stained cells are shown as the insert in (D).

*Bacteriastrum jadranum* cells are weakly silicified, cylindrically shaped and assembled in loose filaments, which are comprised of 4–23 cells in natural samples and up to 40–50 cells in cultures. Each cell contains 7–15 small plastids. Circular valves have diameters of 7–17 µm and are adorned with 7–12 setae (Fig. 1A, B). Setae are smooth, very thin and delicate, perpendicular to the chain axis or irregularly curved, extending in length from 23 to 120 µm (Fig. 2A). The terminal setae emerging from the end valves of the chain are shorter and more curved than the intercalary ones (Fig. 2C). The intercalary setae do not cross or fuse at any point with the setae of the sibling valve, running almost parallel among them, which is different compared to the other known chain-forming *Bacteriastrum* species (see Fig. 1C for comparison with *B. furcatum*). In the original description of the species it was reported that the cells are connected by crossing of setae at a distance equivalent to the diameter of 2–3 cells [5] but our observations do not confirm that notion. No observable direct contact between cells or any other kind of visible mechanical interconnection occurs. Nevertheless, the sibling cells are always regularly organised in loose and flexible chain-like formations, and they do not form random cell aggregates. The true colonial nature of *B. jadranum* is confirmed by the existence of two different types of cells, (i) the heterovalvate terminal cells that have one valve with a central slit-shaped process (Fig. 1A) and the other one lacking it, and (ii) the isovalvate intercalary cells that have both valves without processes (Fig. 1B). This feature is typical of the all colonial *Bacteriastrum* species. The only non-colonial species inside the genus, *B. parallelum*, has a central process on both valves of the cells, which indeed represent terminal valves [6].

In order to detect the existence of an optically transparent matrix presumably keeping the cells in chains we performed staining experiments using Alcian Blue and Coomassie Brilliant Blue G. The experiments with Alcian Blue staining revealed the existence of polysaccharide material enclosing the *Bacteriastrum* cells (Figs. 2B, D). We termed the blue stained structure with a well-defined border toward the surrounding media as the cell jacket. The *Bacteriastrum* jacket was observed in both cultured (Fig. 2D) and field samples (Fig. 2B), indicating that it represents a stable component of the cellular organisation of the species and not an artefact due to culture conditions. A parallel staining experiment with amino acid specific dye Comassie Brilliant Blue G (Fig. 2D insert) showed that substantial amounts of proteins are not present in the structure. The dye stained only cells' interior contents and the bacteria presumably growing on the jacket organic matrix, but the material itself was not stained. The diatoms from the family Chaetocerotaceae are known to produce large amounts of EPS with polysaccharides as the dominant component usually releasing them in the ambient water [9], [10], [11] and these substances are sometimes visible as a mucus sheath around the chain colonies with an optical microscope with water mounts [12]. However, the primary means of association of adjacent cells in these species are always by interlocking or fusion of the siliceous setae and not by cell exudates.

A closer look at the colonies showed that the long and delicate setae were completely enclosed within the jacket domain. The presence of setae in members of the family *Chaetoceraceae* may act as a defence against grazing [13] by injuring or hurting the digestive structures in some grazers, such as the filter-feeding apparatus in appendicularians [14]. However, the *B. jadranum* setae are very delicate and completely enclosed within the polysaccharide jacket; therefore, we argue that their function is not defensive; rather, they represent the mechanical support and stabilisation of the colonial polysaccharide matrix. The *B. jadranum* chain colonies can be compared with the gelatinous colonies of some marine centric diatom species from the genus *Thalassiosira*, the cells of which are embedded in thick mucilage and extrude many chitin threads with the assumed role of reinforcing the organic matrix [15]. The main difference between these *Thalassiosira* and *Bacteriastrum* colonies is that in the former, the colonies appear to be irregular clusters of the morphologically identical cells [16] while in the latter; cells are regularly arranged in chains. Although there are a few *Cyclotella* species in which cells are united in ordered chain colonies without evident contact among valves [17], the essential distinct feature of the *Bacteriastrum* chains remains the existence of the morphologically different end cells of the chain.

### The cell jacket structure at the micro and nanoscale

To resolve the ultrastructure of the cell jacket detected by Alcian Blue staining, we introduced the use of AFM, which uses a sharp probe to image the topography of surfaces at a lateral resolution greater than 1 nm and a vertical resolution of 0.01 nm under ambient conditions [18]. AFM experiments were performed at two quite different scales: 50–100 µm with a vertical scale of 100–1000 nm to identify cell colony and 1–10 µm with a vertical scale of only 5–20 nm to resolve the ultrastructure of overlaid polysaccharide network. All images were acquired in contact mode under ambient conditions using mica as the substrate.

**Figure 3:**
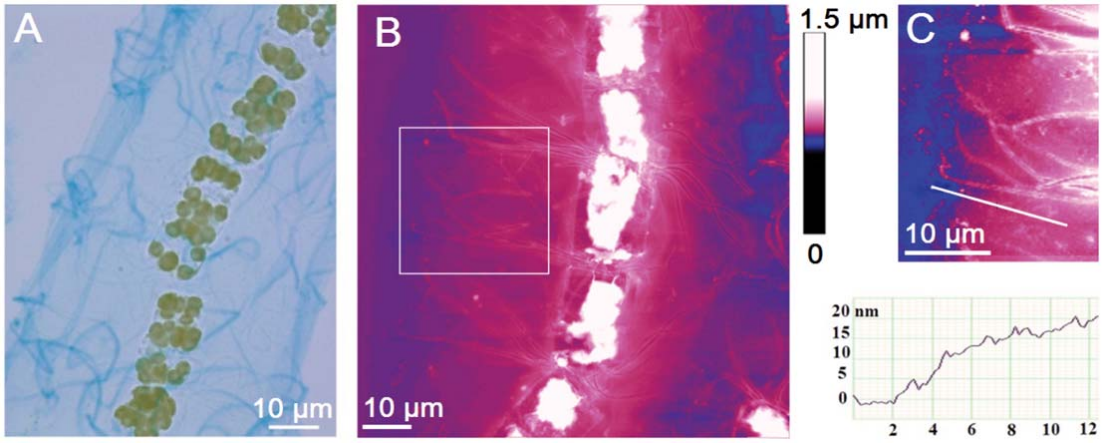
Visualisation of the *Bacteriastrum* cell jacket on a microscale. (A) Light microscopy images of *Bacteriastrum* cell colony from cultured material stained with Alcian blue (phase contrast), (B) AFM image of similar chain presented as height data with vertical scale of 1.5 µm, (C) zoomed view (white box in B) of envelope surrounding cells after maximising the contrast and height analyses along indicating line. Scan sizes of AFM images are 85×85 µm (B) and 30.5×30.5 µm (C).

We started with low magnification (Fig. 3) to identify the *Bacteriastrum* colony – the cells overlaid with a polysaccharide cell jacket and to relate the AFM image to the light micrograph of the cell colony. Thin and fragile, Alcian Blue-stained structure surrounding diatom cells (Fig. 3A) can be directly related to AFM image of a similar cell chain (Fig. 3B). Individual cells, polysaccharide jacket and setae are clearly identified. The cell jacket extends up to 35 µm from the cell centre (30 µm in light micrograph). White areas on the central part of individual cells are the highest zones (700–900 nm) representing cell content and plastids (brown in Fig. 3A). The cell jacket is more clearly recognised at higher magnifications (Fig. 3C). The jacket is a continuous, bulk material reaching the span of setae. The directly measured jacket height in a dry state is only 20–30 nm. For the comparison, the height of the gelatinous polysaccharide capsule of the freshwater green algae *Spondylosium panduriforme* [19] was found to be 130 nm using the same visualisation method, AFM in dry state [20]. The algae capsule material was described as smooth and continuous. The *S. panduriforme* capsule was taken as an essential part for the cell based on its selective permeability studied by electron paramagnetic resonance [21], [22].

**Figure 4:**
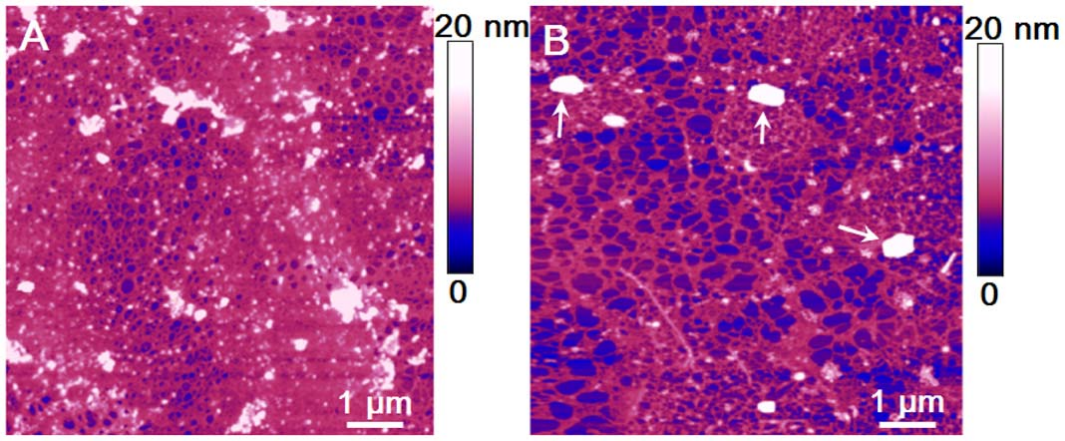
AFM images of the polysaccharide network spread over mica substrate. (A) collapsed 3D network and (B) stretched 2D network. The arrows indicate salt crystallites. Scan size 8×8 µm with vertical scale of 20 nm.

Although AFM imaging showed many details of *B. jadranum* single cell morphology, here we focus on revealing the ultrastructure of the cell jacket. Further on, AFM images of the cell jacket material were captured several hundred nm beyond the observed jacket border. This is the region where the jacket material is spread on the mica substrate as a thin layer, which is an essential feature for high resolution imaging [23]. The cell jacket imaged at 8 µm with a 20 nm vertical scale appeared as a cross-linked fibrillar network (Fig. 4). The observed difference is caused by the extent of spreading over the mica substrate: a collapsed 3D network (Fig. 4A) and a 2D stretched network (Fig. 4B).

**Figure 5:**
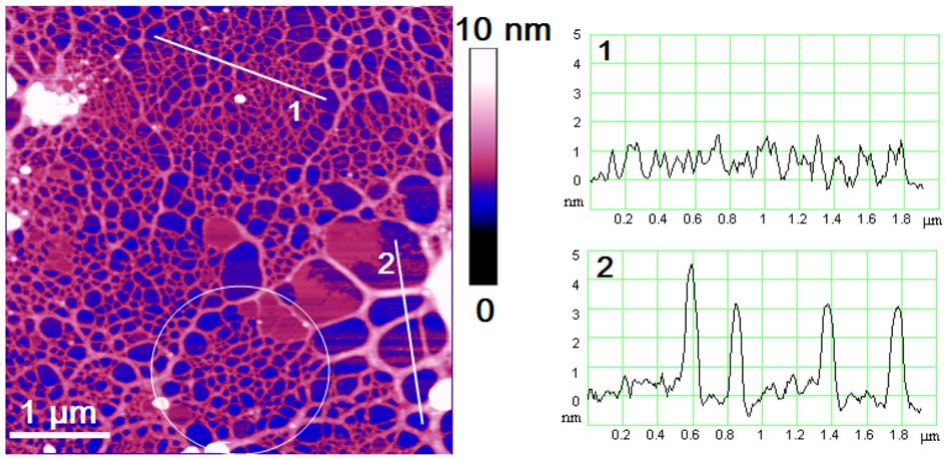
Specific patterns of the cell jacket revealed by high resolution AFM imaging. A high density domain (patch) is encircled in white. Scan size 4.5×4.5 µm with vertical scale of 10 nm. The vertical profiles present the fibrils heights along indicated lines. The white coloured regions (up to 20 nm high) could represent inorganic or organic nanoparticles entrapped from the surrounding media.

By imaging at higher resolution (4 µm and a vertical scale of 10 nm), we detect the specific patterns of the cell jacket network. Frequently encountered 2D fibrillar network patterns are presented in Figure 5 together with height profiles along indicated lines. High-density domains (further called patches) are surrounded and interconnected by thicker fibrils in a continuous network and are taken as the basic structural motive (encircled in white in Fig. 5). Pores inside the patch are of the same hexagonal shape. Their size is continuously smaller from the patch edge towards the centre.

**Figure 6:**
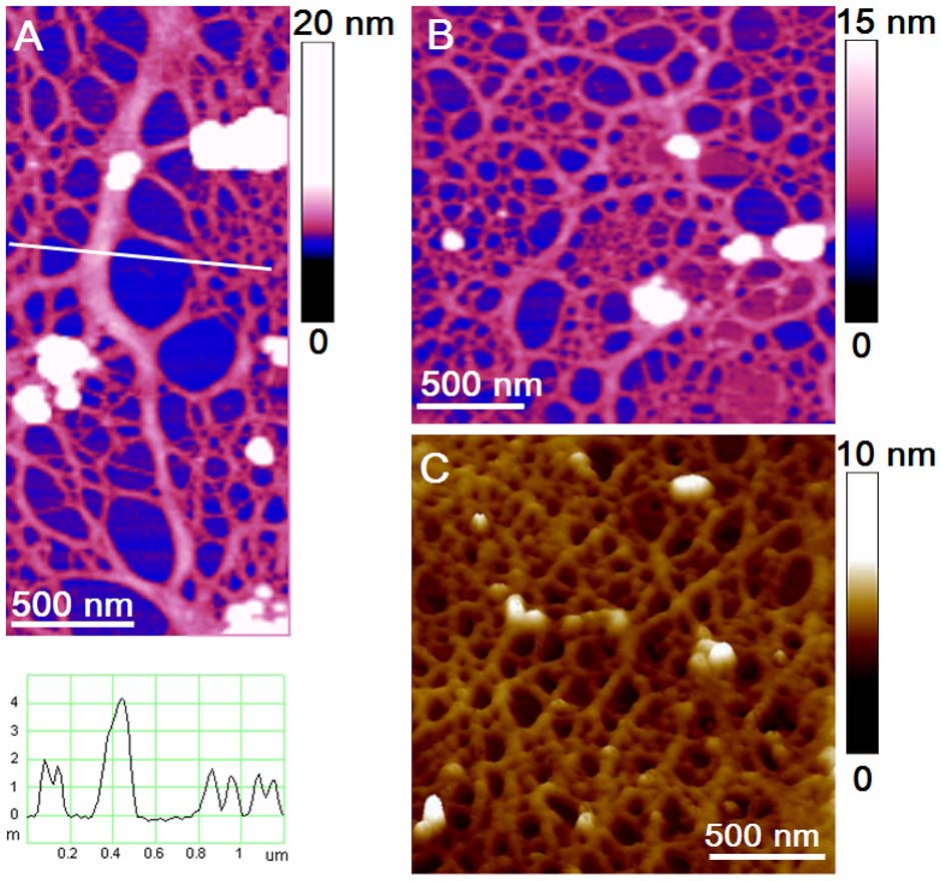
Structural details of the cell jacket network. (A) Branching fibril with height analysis along indicating line; (B) 2D network of interconnect patches; (C) spatial arrangement of interconnect patches in the 3D collapsed network (3D view).

The cell jacket network is reinforced by the fibrils branching into patches (Fig. 6A). Such fibrils are considerably wider but not higher than the fibrils that form large pore openings (height profile in Fig. 5). The branching fibrils are obviously the backbone of the network and critical for assuring its integrity. We succeeded in visualising the interconnected patches, both as a 2D stretched network and as a 3D collapsed network. The self-repeating pattern in 2D network is obvious (Fig. 6B). The spatial arrangement is also discerned as upper and lower layers of 3D network are remarkably well resolved (Fig. 6C).

The collected AFM data, as those presented in Figure 5A, were analysed in terms of fibril heights and pore openings using automatic pore detection image software. The results are presented in Table 1. The pore surface area was converted in pore diameter assuming the pores are circular in shape. The size of the pores may be overestimated due to a stretching of the network upon the deposition on the mica. The lowest measured value of fibril height was found to be 0.6 nm and is taken for the height of a monomolecular fibril. This is in line with monomolecular fibril height for the diatom *Cylindrotheca closterium* EPS [7] and for monomolecular polysaccharide fibrils in marine gel network [23]. Higher values reflect the association of two or more monomolecular fibrils. However, irrespective of the same monomolecular fibril heights and predominantly polysaccharide composition, the fibrils in the marine gel network are not organised in recognisable patterns. The same holds for the *C. closterium* EPS networks. This led us to conclusion that the cell jacket is an essential part of the *Bacteriastrum* cell and not a random extracellular polysaccharide structure supported by the flexible setae.

**Table 1:** Cell jacket network: pore openings and corresponding fibril heights analysed over a surface area of 4×4 µm^2^.

Pore forming fibrils	Pore openings	Number of pores
height/nm	nm	
0.6–1.6	8–100	900
2.0–2.8	100–160	200
2.5–4.0	500–1000	10

**Figure 7:**
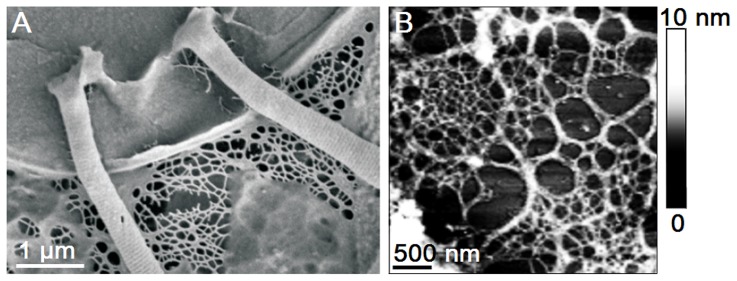
Comparison of the cell jacket fibrillar network obtained with SEM and AFM. (A) SEM images of the *Bacteriastrum* cell with surrounding fibrillar network connected to the cell valve and (B) cell jacket network obtained by AFM (3D view).

This conclusion is further supported by SEM visualisation of the *B. jadranum* polysaccharide network (Fig. 7A). The SEM image represents the partial view of the diatom valve with the two setae and the clearly distinguishable fibrillar organic network associated with the cell. The spatial arrangement of the fibrils and overall appearance of the delicate network structure looks surprisingly similar (Fig. 7A and B), although they were obtained with the two quite different imaging techniques, i.e. electron diffraction and atomic force sensing by a scanning probe. The conjunction of the polymer network with the siliceous cell valve appears to be extremely tight, withstanding extensive preparative procedures needed for SEM imaging. The lack of studies focused on the structural aspect of EPS secreted by the diatoms from the family Chaetocerotaceae does not allow us to compare the ultrastructural features of the *Bacteriastrum* network with the ones produced by the organisms of the similar taxonomic affiliation. However, to our knowledge, such an ordered mosaic-like network of polysaccharide fibrils has not yet been reported in the literature.

## Conclusions

*Bacteriastrum jadranum* cells in colonies are enclosed within an optically transparent organic matrix called a cell jacket. Visualisation of the general layout of the structure with Alcian Blue staining showed the long setae completely enclosed within the jacket and indicated that the jacket's principal components are acidic polysaccharides. The structure of the polysaccharide jacket surrounding the colony must be porous to enable the efficient nutrient transport necessary to maintain cell metabolism and at the same time highly flexible and mechanically stable to ensure the integrity of the chain in the turbulent marine environment. We can argue that using AFM we were able to visualise and characterise such a organisation of the fibrillar polymer network that fulfils these requirements. At the nanoscale, the optically transparent organic matrix appears as a cross-linked fibrillar network organised in recognisable structure. The circular patches of a self-repeating pattern composed of hexagonally shaped pores are connected through thicker surrounding fibrils and reinforced by branching fibrils. The well-defined and regular shape on the microscale, as well as the ordered structure on the nanoscale, indicate that the *B. jadranum* polysaccharide organic matrix mechanically supported by the delicate setae represents an extended, but nevertheless essential, part of the cell. This is further supported by the facts that the conjunction of the polymer network with the frustule appears to be extremely tight and such specific and unique patterns have never been found in self-assembled polysaccharide gel networks usually encountered in the marine environment.

## Materials and Methods

### Ethics statement

No specific permits were required for the sampling as the location is not privately-owned or protected in any way, and the field studies did not involve endangered or protected species.

### Strain isolation and culture maintenance

The material for this study was collected from the Maun Channel (44°28′N, 14°54′E), northeastern Adriatic Sea, Croatia, in October 2010. Observations on *Bacteriastrum jadranum* were made on the cells from both laboratory cultures and natural samples. Phytoplankton samples were obtained from plankton tows (20 µm mesh size), partly preserved through addition of glutaraldehyde (1% final concentration) and partly maintained live until observations in the laboratory. Individual cells or chains were isolated from live net samples in an inverted Zeiss Axiovert 200 light microscope (Carl Zeiss, Oberkochen, Germany) with a micropipette and placed in a 4-well Nunclon Multidish (Nunc A/S, Roskilde Denmark) with 1 mL f/2 culture media [24] diluted 50% with sterile seawater. All wells were observed after some days to check the growth and conditions of cells, and when the cell density was high enough, the cultures were transferred to 50-mL glass Erlenmeyer conical flasks filled with 30 mL f/2 culture media. The clonal cultures were maintained under cool white (40 W) fluorescent lights (30 µmol m^−2^ s^−1^) at room temperature of 20°C in a 16 h∶8 h light/dark cycle and sub-cultured every week.

### Light microscopy and staining procedures

*Bacteriastrum* chain colonies from natural samples were observed in water mounts with Zeiss Axiovert 200 light microscope and images taken using a Zeiss MRc digital camera and processed with AxioVision 4.8.2 digital system (Carl Zeiss, Oberkochen, Germany). Cells from cultures were observed with light microscope Olympus model BX51 equipped with DP70 Digital Camera System and operating with DP Controller and DP Manager software (Olympus Corporation, Tokyo, Japan). Both microscopes were equipped with phase contrast.

Two stains were applied to examine their reactions with the extracellular polymeric substances embedding the cell colonies. Alcian Blue is a cationic dye that stains acid mucopolysaccharides and glycosaminoglycans, and it is commonly used in staining transparent exopolymeric substances in the marine environment [25], [26] [25], [27]. Amino acid specific dye Coomasie Brilliant Blue −250 was used for detection of proteins [28]. A drop of each dye was added directly to the small amount of sample from both glutaraldehyde fixed net sample and laboratory cultures on the glass slides and microscopically observed.

### Atomic force microscopy

All experiments were performed using drop deposition method modified for marine samples [7], [23]. A 5-µL volume of the cell culture was pipetted directly onto freshly cleaved mica. Mica slides were placed in enclosed Petri dish for approximately 30–45 min. Samples were then rinsed three times for 30 s in ultrapure water and placed in enclosed Petri dish to evaporate excess water from the mica. All measurements were performed in air at room temperature and 50–60% relative humidity, which leaves the samples with a small hydration layer, helping to maintain the original structures.

AFM imaging was performed using a Multimode AFM with a Nanoscope IIIa controller (Bruker, Billerica USA) with a vertical engagement (JV) 125-µm scanner. Contact mode was used throughout the study. Imaging was performed using standard silicon-nitride tips (NP-20, Bruker, nom. freq. 56 KHz, nom. spring constant of 0.32 N/m) and extra sharpened silicon nitride tips (MSNL, Bruker, tip radius nom. 2 nm, nom. freq. 4–10 KHz, nom. spring constant of 0.01 N/m) for high-resolution imaging. The force was kept at the lowest possible value to minimise the forces of interaction between the tip and the surface. Continuous scans were performed over the same region (10 times, slow scan: 1 Hz, 512 samples), and the structures were not altered. The linear scanning rate was optimised between 1.5 and 2 Hz with scan resolution of 512 samples per line. Processing and analysis of images was carried out using NanoScope™ software (Digital Instruments, version V614r1). All images presented are raw data except for the first order two-dimensional flattening. Analysis of pore size was performed by automatic pore detection and pore surface measurements using Image J software.

### Scanning electron microscopy

To remove organic matter, samples from cultures of *Bacteriastrum furcatum* and *B. jadranum* were prepared using Simonsen's method (KMnO_4_ and HCl) for cleaning diatom frustules [29]. The cleaned material was filtered on a 3-µm Nucleopore polycarbonate filter. The glutaraldehyde fixed net sample containing the chain colonies of *B. jadranum* was directly filtered on the polycarbonate filter and rinsed with distilled water. The sample was dehydrated in a series of ethanol solutions (25, 35, 50, 75, 80, 90%) prepared with distilled water and absolute ethanol, finishing with three rinses of 100% ethanol. For the drying method, the hexamethyldisilazane (HMDS) treatment was used [30]. The sample was rinsed in a series of 100% ethanol: HMDS solutions (3∶1, 1∶1, 1∶3), finishing with three changes of 100% HMDS. The sample was treated for a minimum of 5 min at each step, allowing the last HMDS rinse to evaporate slowly at room temperature. Both prepared filters, with cleaned and non-cleaned samples, were mounted on stubs, sputter coated with gold and examined using a JEOL JSM-6500F scanning electron microscope (Peabody, MA, USA).
